# Food subsidy programs and the health and nutritional status of disadvantaged families in high income countries: a systematic review

**DOI:** 10.1186/1471-2458-12-1099

**Published:** 2012-12-21

**Authors:** Andrew P Black, Julie Brimblecombe, Helen Eyles, Peter Morris, Hassan Vally, Kerin O′Dea

**Affiliations:** 1 Sansom Institute for Health Research, Division of Health Sciences, University of South Australia, Adelaide, South Australia, Australia; 2 Menzies School of Health Research, Charles Darwin University, Darwin, NT, Australia; 3 National Institute for Health Innovation, School of Population Health, University of Auckland, Auckland, New Zealand; 4 School of Public Health and Human Biosciences, La Trobe University, Melbourne, VIC, Australia

**Keywords:** Food subsidy, Disadvantaged families, Health outcomes, Dietary intake, Nutritional status

## Abstract

**Background:**

Less healthy diets are common in high income countries, although proportionally higher in those of low socio-economic status. Food subsidy programs are one strategy to promote healthy nutrition and to reduce socio-economic inequalities in health. This review summarises the evidence for the health and nutritional impacts of food subsidy programs among disadvantaged families from high income countries.

**Methods:**

Relevant studies reporting dietary intake or health outcomes were identified through systematic searching of electronic databases. Cochrane Public Health Group guidelines informed study selection and interpretation. A narrative synthesis was undertaken due to the limited number of studies and heterogeneity of study design and outcomes.

**Results:**

Fourteen studies were included, with most reporting on the Special Supplemental Nutrition Program for Women, Infants and Children in the USA. Food subsidy program participants, mostly pregnant or postnatal women, were shown to have 10–20% increased intake of targeted foods or nutrients. Evidence for the effectiveness of these programs for men or children was lacking. The main health outcome observed was a small but clinically relevant increase in mean birthweight (23–29g) in the two higher quality WIC studies.

**Conclusions:**

Limited high quality evidence of the impacts of food subsidy programs on the health and nutrition of adults and children in high income countries was identified. The improved intake of targeted nutrients and foods, such as fruit and vegetables, could potentially reduce the rate of non-communicable diseases in adults, if the changes in diet are sustained. Associated improvements in perinatal outcomes were limited and most evident in women who smoked during pregnancy. Thus, food subsidy programs for pregnant women and children should aim to focus on improving nutritional status in the longer term. Further prospective studies and economic analyses are needed to confirm the health benefits and justify the investment in food subsidy programs.

## Background

Good nutrition is an important factor in maintaining and promoting health, particularly given the global rise of non-communicable diseases such as vascular diseases and cancer in most countries. The Global Burden of Disease studies estimated that nutrition-related risk factors such as overweight/obesity, high cholesterol, inadequate fruit and vegetable intake and high blood pressure cause 25% of disease and disability each year [1]. In addition, nutrition has a vital role in pregnancy and early childhood. During pregnancy, good nutrition is especially important in supporting fetal development and protecting the mother from pregnancy-related risks, such as gestational diabetes, excessive weight-gain, pregnancy-induced hypertension and iron-deficiency anaemia [2]. Nutrition complements other factors in early childhood which promote development and foster healthy behaviours that hopefully travel into adulthood.

International research suggests that there are multiple barriers to improving people′s nutrition including the cost and taste of healthy food, poor dietary habits, and a limited understanding of nutritional concepts [3-5]. In high income countries, there has been increasing focus on the increased intake of energy-dense nutrient poor foods by a majority of the population [6-8]. However, low socioeconomic status is associated with lower uptake of health promoting behaviours [9], including healthy eating [10,11]. For those on low incomes in high income countries, the cost of healthier food is considered an important barrier to improving the quality of dietary intake [12,13]. In this context, health promotion and education alone may have little impact in disadvantaged families. Further, structural/ecological interventions, such as food pricing strategies, may have a greater impact on health behaviours than individual interventions alone [14].

Food subsidy programs are one element of food pricing strategies and have been operating for many years in the United States and the United Kingdom. The Special Supplementary Nutrition Program for Women, Infants and Children (WIC) in the United States commenced in 1972 to provide healthy foods, referrals to health and social services and nutrition education to pregnant women and families with young children [15]. The WIC program was developed to target common nutritional deficiencies in the diets of disadvantaged pregnant women and children [16]. It has been evaluated more than any other food subsidy program, with many studies demonstrating improvements in pregnancy outcomes and children′s nutritional status and early intellectual development [17]. However, the rapid uptake of the program nationally made it difficult to conduct robust randomised trials resulting in concerns about selection bias in existing evaluation studies [18-20]. The longstanding Welfare Food Scheme program in the United Kingdom was expanded and renamed Healthy Start in 2006 with a renewed focus on improving the nutrition of low income women and their children [21]. Both WIC and Healthy Start have changed recently to provide more fruit and vegetables (F&V) to address current nutritional challenges in target populations. Food subsidy programs remain topical in other countries; South Korea has trialled a program modelled on the WIC program [22] and the Food Miles program in Canada provides subsidies to wholesale distributors sending perishable foods by air to remote communities, which has reduced the cost of healthy foods for families in remote locations [23]. A collation of existing evidence may contribute to existing and future initiatives in this area.

The only systematic review of food subsidy programmes to date has focussed exclusively on pregnancy-related outcomes [24]. In that 2006 review, D^′^Souza et al. concluded that food subsidy programs can increase key targeted nutrients, but that there was limited evidence of their impact on birthweight or other pregnancy outcomes. It was suggested that the aims of food subsidy programs should be broadened to recognise the need for longer-term support of families throughout child-rearing. The authors also identified the need for robust evaluation of new or expanded programs, such as the Healthy Start Program.

The overall aim of the current review was to determine the impact of food subsidy programs on the nutritional intake and health status of disadvantaged adults and children in high income countries. A secondary aim was to identify any adverse effects of these programs. In order to expand the work of D′Souza et al. [24] both adults and children living in the community were included, while recognising the importance of pregnancy and early childhood. In the context of the global campaign to increase fruit and vegetable consumption [25], the current review considered whether the impact of food subsidy programs on fruit and vegetable intake are sufficient to contribute to reducing non-communicable disease prevalence. Finally, this review focused on high income countries as it is considered that the underlying nutritional status and the community resources available locally are distinctly different in low and middle income countries.

## Methods

This review was undertaken based on the methods outlined in the Cochrane Handbook [26] and the Cochrane health promotion and public health guidelines [27].

### Criteria for studies included in the review

#### Types of studies

Randomised controlled trials (RCTs), controlled before- and after studies (CBAs), and interrupted time series analyses of routine data (ITS) were eligible for inclusion. ITSs were defined as analyses where data had been collected at three or more time points both before and after an intervention was implemented.

#### Types of participants

Eligible participants were socio-economically disadvantaged adults, children or families living independently in the community in high income countries. High income countries were as defined by the World Bank [28]. Special population groups such as the homeless or those in substance abuse treatment programs were excluded.

Socio-economic disadvantage was defined as

• families/participants from areas described as disadvantaged by authors (e.g. low income area, ghetto, social housing projects); or

• families/participants of low socio-economic status (SES) (e.g. working class, low income, unemployed); and/or

• disadvantaged minorities e.g. Indigenous peoples.

#### Types of interventions

Eligible interventions were those that provided subsidized food alone or in combination with other health interventions. The food subsidy programs may have used any direct or indirect strategy to reduce the price of food including policy initiatives, transport and infrastructure subsidies, cross-subsidies. Examples of transport and infrastructure subsidies include the Food Miles program [23] in Canada which subsidises wholesale food transportation costs to remote communities and the Outback Stores initiative [29] in Australia which assists remote Indigenous community stores to improve infrastructure and storage processes to reduce the costs of perishable foods. Eligible food subsidy programs had to have provided a 10% or greater reduction in the price of targeted foods because this level of reduction has been shown to impact on food expenditure [30,31]. Interventions which provided pre-prepared meals to participants (e.g. elderly or frail) were excluded.

In the RCTs and CBAs, at least one group had to have received a food subsidy program and another group to serve as a control (no intervention, delayed intervention, or attention control). Both intervention and control groups in eligible studies may have also received nutrition education/promotion. In addition to studies of the standard WIC program, there was one CBA study which compared the addition of F&V to the standard WIC program (prior to the inclusion of F&V) [32].

Emergency food relief services (eg Food banks) were excluded as they provide intermittent or one-off assistance. Given the challenge of changing food habits, it is unlikely that such intermittent nutrition interventions would have sustained impacts on nutritional intake. School meals programs were also excluded as these programs were reviewed in a recent (2007) Cochrane review [33]. This review was designed to focus on interventions aimed at families in the community, as these were considered to be distinct from school-based nutrition interventions.

#### Outcomes

To be eligible for the review, a study must have reported validated measures of at least one of the following as a primary outcome:

• Nutritional intake/food purchases (measured by validated dietary assessment techniques, food purchasing, or biomarkers)

• Anthropometric measures-e.g. body weight, body mass index, waist circumference

• Any measure of physical health- e.g. mortality, morbidity, hospital admissions or emergency department attendances

• Pregnancy-related outcome measures- e.g. rate of pre-term delivery, or low birth weight (LBW), mean birth weight

• Child growth and development measures e.g. BMI percentile for age

• Health service utilization- e.g. vaccination rates, participation in preventive health activities

Any adverse outcomes reported for participants were recorded (e.g. stigmatisation, dependency, decreased total food expenditure, increase in high fat/high sugar foods (including take-away food) and obesity or excessive weight loss).

#### Search strategy

The following electronic databases were searched for relevant articles published between 1980 to November 2010:

Medline, Central (Cochrane), DARE, Embase, Cambridge Scientific Abstracts - Social Services Abstracts & Sociological Abstracts, Web of Science- Science Citation Index, Social Science Citation index, CINAHL, Informit- Health, Food Science and Technology Abstracts, and EconLit.

A Medline search strategy was developed that incorporated terms for: 1.Food (eg health food, fruit, vegetables, food preferences, food habits), 2.Nutrition programs (eg. food subsid*, national health program, price discount*, nutrition policy, fruit or vegetable subsidy) and 3.Nutrition and health outcomes (eg. outcome assessment, nutritional physiological phenomena, biological markers, health behaviour, nutritional status, carotenoids). Filters for high income countries and study design, adapted from another systematic review of community level interventions to improve food security [34], were applied. The Medline search strategy was developed and then adapted for other databases as required. These are available from the authors upon request. Bibliographies of screened studies and relevant reviews and manuscripts were also searched for eligible studies and a search was conducted of the following relevant websites: The Food and Nutrition Service (http://www.fns.usda.gov/fns/) and the Economic Research Service (http://www.ers.usda.gov/) of the United States Department of Agriculture.

#### Data synthesis and analysis

All manuscripts from searches were downloaded into an Endnote library. The titles were scanned by two authors independently (JB, AB). The abstracts of potentially eligible studies were then assessed by one author (AB). Full-text manuscripts for potentially eligible studies were obtained and reviewed by two authors using the eligibility criteria (JB, AB). These two authors achieved consensus on the eligibility of all studies after discussion of any discrepancies. Records of reasons for rejection were kept. Data was extracted into a standard template adapted from the Cochrane Handbook [26] and the EPOC data collection checklist [35] by one author (AB). Data entry was checked for each study after completing data extraction. All primary outcomes, any adverse outcomes, together with age, gender, pregnancy status and cultural background of study population, the setting for each study and details of the study design were extracted. The study authors were contacted to try and obtain missing data.

Due to the heterogeneity of study designs and outcomes and the limited number of studies reporting individual outcomes, narrative synthesis was used to summarise the majority of outcomes. The recommendations of the Cochrane Handbook were used to assess the adequacy and appropriateness of undertaking meta-analysis for each outcome [26].

#### Assessment of risk of bias in included studies

Included studies were assessed for risk of bias on relevant domains based on the Cochrane guidelines for RCTs [26] and both the Effective Practice of Care (EPOC) guidelines [35] and the Newcastle-Ottawa Scale [36] to assess the quality of CBA and ITS study designs. In accordance with Cochrane guidelines [26], the ratings for each domain are summarised in Additional file 1. Studies were assessed as low risk of bias if there were 0–1 criteria not met, moderate risk of bias if up to two criteria were unclear and high risk of bias if 2 or more criteria were not met (Table 1).

**Table 1:** Characteristics of included studies (in chronological order)

**First author, year, Setting/Location**	**Participants, Recruitment**	**Interventions, No. of subjects**	**Methods**	**Outcomes**	**Duration of intervention**	**Potential risk of bias rating**
**Bailey 1983**[48]	101 pregnant women aged 15–41 years	1. Standard WIC program- Monthly vouchers for specified quantities of milk, canned fish, carrots, cereal, cheese, eggs. 6 monthly nutrition education and health care referrals n=43	Controlled before and after study	Serum iron, vitamin B6, folic acid,	12 weeks	High
WIC clinic and hospital prenatal clinic, Florida, USA	WIC and control participants recruited from different prenatal clinics at 30 weeks gestation			Red cell folate		
		2. Routine antenatal care through hospital clinic n=58	Follow-up period 12 weeks	Dietary iron, vitamin B6, and folic acid		
				Birth weight		
				% Low birth weight		
**Metcoff 1985**[42]	824 pregnant women stratified by predicted birth weight; all WIC eligible	1.Standard WIC program with research assessments and routine prenatal care n=238	Randomised controlled trial	Birth weight	~21 weeks	High
				Plasma B-carotene		
Hospital prenatal clinic, Oklahoma City, Oklahoma, USA				Maternal weight		
	All participants recruited from same hospital prenatal clinic	2. Routine prenatal care with research assessments n=172	Follow-up period 24 weeks	Plasma amino acids		
		3. Routine prenatal care n=353				
**Caan 1987**[38]	703 post-partum women- all WIC participants prenatally	1. Standard WIC program maintained for 6 months post-partum for non-lactating women n=333	Controlled before and after study	Birth weight	6 months	High
				Low birth weight		
				Macrosomia		
48 local WIC agencies, California, USA	All WIC participants prenatally, divided into control &intervention groups retrospectively based on WIC benefits post-partum	2. Standard WIC entitlement for 0–2 months post-partum n=309		Maternal Hb		
			Follow-up period-duration of prenatal care in second pregnancy	Maternal BMI		
**Rush 1988a**[44]	11,154,673 pregnant women from 1392 US counties in 19 states	1. Standard WIC program	Interrupted time series	Birth weight	Duration of prenatal WIC participation	Low
				Duration of gestation		
National sample of counties, USA	WIC participants increased over time from 0 to 39% from government reports		Follow-up period 1972-1981	Fetal mortality (>28 weeks)		
				Infant mortality rate		
				Inadequate prenatal care		
**Rush 1988b&c**[45,47]	6563 pregnant women, all WIC eligible by income	1. Standard WIC program and research assessments n=5205	Controlled before and after study	Nutrient intake	Duration of prenatal WIC participation	High
174 WIC clinics and 55 prenatal clinics, national sample, USA	WIC participants recruited at WIC clinics, controls recruited at public prenatal clinics in counties with low WIC coverage	2. Routine antenatal care and research assessments n=1358	Follow-up period 6–9 months			
				Mean nutrient intake % RDA		
				Anthropometry		
				Duration of gestation		
				Birth weight		
				Fetal mortality		
**Rush 1988c&d**[45,46]	5004 pregnant women, mean age 22.4 years	1. Standard WIC program n=4219	Controlled before and after study	Family food expenditure	Duration of prenatal WIC participation	High
174 WIC clinics and 55 prenatal clinics, national sample, USA		2. Routine antenatal care n=785		Family grocery expenditure		
	WIC participants recruited at WIC clinics, controls recruited at public prenatal clinics in counties with low WIC coverage	A subset of women were asked to complete a food diary at follow-up: WIC n=1031, Control n=551	Follow-up period 6–9 months	Family meals out expenditure		
**Gunnell 2000**[39]	1089 children aged 2–14 years, mean age 8 years	1. Daily school feeding soup/milk, halibut oil capsules, oranges or milk and marmite n=298	Controlled before and after study	Height	12 months	Moderate
				Leg length		
8 rural and urban locations in Scotland and England, UK	Disadvantaged families selected and divided into intervention and control groups arbitrarily					
		2. Family food packages weekly-milk, cheese, wheat germ, marmite, oranges, cod liver oil, eggs n=269	Follow-up period 13 months for children′s growth and 60 years for mortality	Mortality		
	School in adjacent areas were also selected as intervention and controls non-randomly	3. No food subsidy- control families n=261				
		4. No food subsidy- control schools n=258				
**Pehrsson 2001**[43]	110 post-partum non-lactating females >18 years old, all WIC participants prenatally	1. Standard WIC program for 6 months post-partum n=57	Controlled before and after study	Haemoglobin	6 months	Low
				Transferrin receptor		
Urban WIC clinics, Maryland, USA	Participants recruited at WIC clinics in different counties	2. Standard WIC program for 0–2 months post-partum n=53	Follow-up period 6 months	Anaemia %		
				Ferritin		
**Burr 2007**[37]	190 pregnant females aged >=17 yo	1. Vouchers for free 2L fruit juice weekly (home delivery) n=63	Randomised controlled trial	Frequency of specific fruit consumption	Throughout prenatal care period (~30 weeks)	High
District hospital prenatal clinic, Wales, UK (disadvantaged area)	All participants recruited from one hospital prenatal clinic at booking visit	2. Advice/written information from midwives to promote fruit intake during pregnancy n=63	Follow-up period 30 weeks	Serum β-carotene		
		3. Routine antenatal care n=64				
**Herman 2006 & 2008**[32,40]	602 post-partum women >18yo**-** all WIC participants	1. $10 voucher weekly for F&V at local supermarket plus standard WIC program n=200	Controlled before and after study	F&V intake	6 months	High
3 WIC clinics, Los Angeles, California, USA	Intervention and control participants recruited at three separate WIC clinics with similar socio-demographics	2. $10 voucher weekly for F&V at local farmers market plus standard WIC program n=200	Follow-up period 1 year			
		3. Standard WIC program n=202				
**Currie 2008**[49]	All pregnant Californian women 1961–1974 n=4864673	1. Standard Food Stamp Program- monthly food vouchers for any foods up to $200/person/month dependent on income and household size	Interrupted time series	Median birthweight	Duration of prenatal FSP participation	Low
				Low birthweight rate		
California, USA	Food stamp participation rates by county used to calculate impacts		Follow-up period 1961-1974	Probability of birthweight <specified cut-off		
**Hoynes 2009**[18]	28,000,000 pregnant women in the 2059 US counties with a WIC program by 1979 (85% of US births in the 1970s)	1. Standard WIC program	Interrupted time series	Birth weight	Duration of prenatal WIC enrolment	Low
National sample of counties, USA			Follow-up period	% Low birth weight		
	WIC participation estimated from government reports		1971-75 & 1978-82			
**Kennedy 2009**[41]	40 African-American women aged >18yrs, non-pregnant	1. F&V $10/week with recipes from mobile store at community centre, monthly nutrition education, cooking demonstrations and anthropometric assessment n=20	Randomised controlled trial	Fruit and vegetable consumption	6 months	High
Community centre, East Baton Rouge, Louisiana, USA	Participants recruited by local community advertising and personal communication	2. Monthly anthropometric assessment and written nutrition education n=20	Follow-up period 6 months	Weight		
				BMI		
				BP		
				Quality of life		
				General and emotional health		
**Ni Mhurchu 2010**[30]	1104 adult >18 years, main household shopper, 86% female	1. Tailored nutrition education– computer-generated messages and shopping lists plus generic recipes monthly by mail n=274	Randomised controlled trial	Total food purchased	6 months	Low
8 supermarkets in Wellington, Wanganui and New Plymouth, NZ			Follow-up period	Healthy food purchased		
	Participants recruited by mail invitation, supermarket advertisements and community group promotion (for Maori and Pacific communities)	2. Price discount of 12.5% on healthy foods applied automatically at check-out n=275				
			15 months	Less healthy food purchased		
				F&V purchased		
		3. Price discount plus tailored nutrition education n=277		Macronutrients purchased		
		4. Control n=278				

## Results

**Figure 1:**
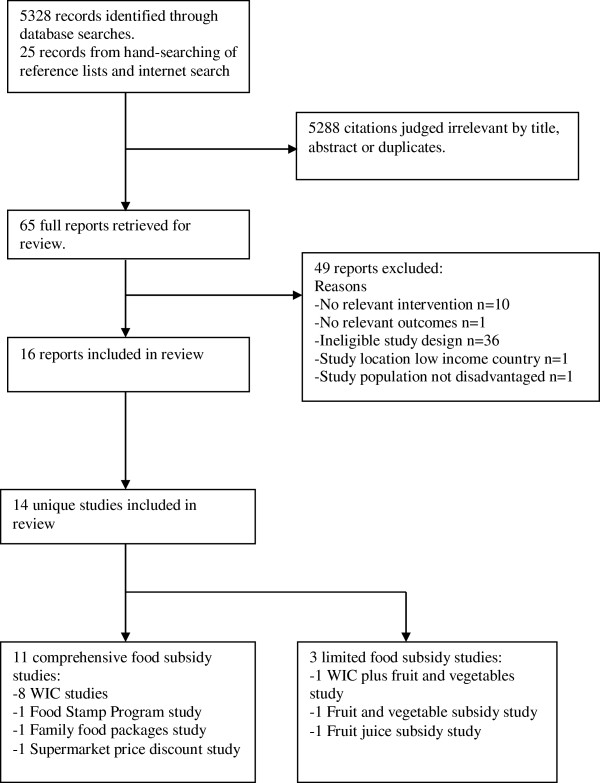
Flow-chart of search results.

Systematic literature searching identified 5,328 articles from which 684 were retrieved for abstract review (see Figure 1). There were 16 articles (14 separate studies) which met the inclusion criteria and were included in the systematic review [18,30,32,37-49].

The 14 studies included four RCTs [30,37,41,42], seven CBAs [32,38,39,43,46-48] and three ITSs [18,44,49]. Eleven of the studies were from the United States [18,32,38,40-49] with nine of these focussed on the WIC program [18,32,38,40,42-48]. Two studies were from the United Kingdom [37,39] and one from New Zealand [30]. The dates of publication ranged from 1983–2010. The characteristics of included studies are shown in Table 1.

Participants in eight of the 14 studies were pregnant women (six WIC [18,42,44,46-48] and two other studies [37,49]). There were also three WIC studies involving postnatal women [32,38,43]. Of the other non-WIC studies there was one study of healthy adults [30] and another of overweight African-American women [41]. There was also one study of 2–14 year old children [39]. The vast majority of participants were low income, from disadvantaged local areas or from Indigenous or ethnic minority groups.

### Scope of included studies

Of the nine WIC studies, there were two ITS studies of routine birth registry data including 28 million births [18] and 11 million births [44]. There was one RCT [42] and two larger CBA studies [46,47] that reported the impact on pregnant women. These larger CBA studies were part of a National WIC evaluation in the 1980s. There were also four smaller CBA studies [32,38,43,48], involving 101–702 participants, which evaluated different aspects of the WIC program. Of the five non-WIC studies, there were three RCTs: one of supermarket price discounts for 1104 regular adult shoppers in New Zealand [30], one of subsidised fruit juice for 190 pregnant women in the UK [37] and another of a F&V subsidy for 40 overweight women in the USA [41]. There was an ITS using Californian birth registry data to evaluate the Food Stamp Program in the USA [49] (renamed the Supplemental Nutrition Assistance Program in 2008) and a 1930s CBA study of supplemental feeding of 1089 children either with family food packages or at school in the UK [39].

### Risk of bias in included studies

The majority (n=9) of the studies appeared to be at moderate to high risk of bias. The studies that satisfied all or most methodological criteria (0–2 relevant criteria not satisfied) and appeared to be at lower risk of bias included three WIC studies- the two ITS [18,44] and a CBA study of postnatal women [43], and three non-WIC studies- the RCT from NZ [30], the ITS of the Food Stamp Program [49] and the CBA study of supplemental feeding of children from the UK [39] (Table 1). Sensitivity analysis using only studies judged to be at low risk of bias was not possible due to the limited number of studies. However, the results from these studies are highlighted in the results and/or discussion. Selection bias was a particular concern in the National WIC evaluation CBA studies; controls were less disadvantaged than WIC participants in these studies and 25% of controls were also found to be on the WIC program at follow-up [45].

### Interventions and Outcomes

The interventions included the standard WIC program [18,42,44,46-48] or enhancements to the WIC program with either an additional six months of postnatal supplements [38,43] or $US10 F&V per week [32]. The standard WIC program includes monthly food vouchers, nutrition education and healthcare referrals at WIC clinics. The food vouchers are for designated quantities of foods including iron-fortified cereal, vitamin C-rich fruit and/or vegetable juice, eggs, milk, cheese, peanut butter, dried beans or peas, canned tuna and carrots (prior to 2009). In 2009, US$10 fruit and vegetables, whole-wheat bread and alternative canned fish were added to packages. Women may receive WIC foods while pregnant and for 12 months if breast-feeding or six months if not breast feeding and children receive foods up to age four years. Non-breast fed infants receive infant formula and all infants are eligible for baby foods from four months [50]. Of the non-WIC studies, interventions included: a 12.5% supermarket price discount on healthier options with or without nutrition education; [30] subsidised monthly food vouchers up to US$142/month (1974 figures) dependent on income (Food Stamp Program in USA); [49] free home-delivered fruit juice weekly; [37] specified family food packages weekly or daily school feeding; [39] and US$10 F&V weekly with nutrition education at a local community centre [41]. The duration of the interventions ranged from 12 weeks for one of the WIC studies [48] to 12 months for the family food packages [39] (Table 1).

The presentation of the impact of food subsidy programs on primary outcomes distinguishes between comprehensive food subsidy interventions which subsidise a wide range of food items or an overall package of food (Tables 2,3,4) and those in which only fruit and vegetables or juice are subsidised (Table 5). This reflects that subsidising a range of healthier foods may be more likely to impact on overall dietary intake, but also that increasing intake of fruit and vegetable intake has become an important public health goal given its the potential to reduce the risk of non-communicable diseases. The comprehensive food subsidy studies include all except one of the WIC studies [18,38,42-44,46-48], the supermarket price discount RCT [30], the family food packages study in the UK [39] and the Food Stamp Program ITS [49]. The fruit and vegetable subsidy studies include the home-delivered fruit juice [37] and the subsidised fruit and vegetables at the community centre [41] and fruit and vegetables as an enhancement of the standard WIC program [32].

**Table 2:** Nutritional and health outcomes associated with comprehensive food subsidy programs for pregnant/postpartum women

**First Author, Year Intervention, Participants**	**Nutritional outcomes**				**Health outcomes**			
**Bailey 1983**[48]	**Dietary intake**				**Perinatal outcomes**			
Standard WIC program	Mean (SD)	I	C	p	Mean (SD))	I	C	p
	Iron, mg	17 (10)	16 (6)	NS	Birth weight, g	3229 (546)	3276 (563)	NS
	Vitamin B6, mg	1.4 (1.1)	1.1 (0.7)	NS	Low birth weight, %(<2500g)	5	10	NS
	Protein, g	90 (39)	105(39)	<0.05				
Pregnant women	Folic acid, μg	264 (216)	239 (159)	<0.05	Ponderal index, g/cm [3]	2.6 (1.2)	2.4 (0.3)	NS
	Energy, kcal	2390 (916)	2496 (879)	<0.05	(Infant weight x100/length [3])			
	**Biomarkers**				**Birth weight**			
					Smokers, g	3286 (515)	2976 (596)	
	Mean (SD)	I	C	p	Non-smokers, g	3218 (538)	3461 (520)	
	Serum iron, ug/dL	106 (44)	99 (42)	NS	p<0.05 for smoking status of control groups only			
	PlasmaVit. B6, ng/ml	4.6 (6.8)	3.3 (1.8)	NS				
	Transferrin sat. %	37 (23)	23 (10)	<0.05				
	Serum folate, ng/ml	14 (11)	26 (26)	<0.05				
	RBC folate, ng/ml	353 (278)	602 (321)	NS				
	Haematocrit, %	35 (3)	35 (3)	NS				
**Metcoff 1985**[42]	**Biomarkers**				**Perinatal outcomes**			
	Mean (SD)	I	C	p	Mean	I	C	p
Standard WIC program	Leukocyte protein synthesis**							
Pregnant participants	pMol 3H-leucine/h	126.6 (33.2)	115.2 (34.3)	0.009	Birth weight, g*			
Results from 410 mother-baby pairs with complete data available					All births†	3254	3163	0.040
	Alanine**	334 (68.4)	350 (70.2)	0.046	Smokers >10 cig/day	3234(n=68)	3059 (n=53)	0.017
	Cystine**	68 (13.7)	72 (11.7)	0.001	Low birth weight, %	8.7	6.9	0.40
	**Adjusted for week of gestation for initial measurement, initial value, elapsed interval between measurements				**Maternal outcomes**			
					Maternal wt gain, kg	16.1	14.7	0.19
					Biceps skin fold, mm	16.2	14.7	0.059
					*Adjusted for gestational age, sex of baby, prenatal care, smoking, interval since last pregnancy, race, history of previous LBW baby			
					†After adjusting for maternal weight at entry to study, the effect of WIC on all births was not statistically significant			
**Caan 1987**[38]					**Perinatal outcomes**			
					Mean (SE)	I	C	p
					Birthweight, g*	3468 (30.0)	3337 (31.1)	0.003
WIC program maintained for 6 months post-partum for non-lactating women instead of normal 0–2 months					Ponderal index,002A g x 100/cm3	2.72 (0.03)	2.73 (0.02)	NS
					Low birth weight, %	3.2	5.1	0.08
					Macrosomia, OR	(95% CI) (I vs C)	1.30 (0.70-2.42)	NS
Pregnant women in subsequent pregnancy					**Maternal outcomes**			
					Mean (SE)	I	C	p
					Hb, g/dL**	12.43 (0.08)	12.14 (0.08)	0.02
					Low Hb, OR (95%CI) (I vs C)	0.65	(0.45-1.07)	0.07
					Mat. de Quetelets index lb/in [2] x100†	3.43 (0.36)	3.59 (0.36)	0.003
					*Adjusted for parity, pregravid weight/height, infant sex, birth weight of last infant, race and smoking status			
					**Adjusted for race, parity, BMI, duration of gestation at time of measurement and anaemia status during 1^st^ pregnancy			
					† Adjusted for race, age, interbirth interval, birth weight of first infant, weight status in first pregnancy, smoking status			
**Rush 1988a**[44]					**Perinatal outcomes**			
					Mean	All births	WIC births	p
Standard WIC program					Birth weight, g	3335	3358	<0.01
Pregnant women					Low birth weight (<2500g), %	6.84	6.41	NS
					Fetal mortality, >28 wk gest/1000	6.21	4.09	NS
					Infant mortality/1000			
					0-27days, total	10.59	8.30	NS
					28-364 days	3.77	4.46	NS
					Duration of gestation, weeks	39.06	39.26	<0.05
**Rush 1988b,c**[45,47]	Dietary intake*				Perinatal outcomes*			
	Mean	I	C	p	Mean	I	C	p
	Protein, g	80.76	75.54	<0.01	Birth weight, g	3292	3285	NS
					Low birth weight (<2501g), %	5.62	6.75	NS
Standard WIC program	Calcium, mg	1003.7	871.0	<0.001	Duration gestation, days	279.0	279.3	NS
	Iron, mg	17.22	14.06	<0.001	Preterm births, %			
Pregnant women	Vitamin A, mg	2.06	1.83	NS	< 33 weeks	0.30	0.90	<0.05
	Vitamin C, mg	134.11	111.68	<0.001	< 37 weeks	9.45	12.07	NS
	Other macro- and micronutrients had statistically significant increases in WIC participants including energy, carbohydrate, fat, magnesium, phosphorus, vitamins B1, B2, B3, B6, B12				Head circumference, cm	34.13	33.95	<0.05
					Fetal mortality/1000	5.09	9.54	NS
	Nutrient intake as % of RDA*							
	Mean	I	C		*Adjusted for duration of gestation and 35 other maternal characteristics including smoking status, age, race, family income and size, woman and partner′s educational and employment status, social security benefits/program participation			
		(%)	(%)		Maternal outcomes*			
	Energy (2400kcal)	84	79		Mean	I	C	p
	Protein (74g)	109	102		Initial weight, kg	65.17	65.89	<0.05
	Calcium (1200mg)	84	73		Follow-up weight, kg	72.17	72.17	NS
	Magnesium (450mg)	60	54		(36 weeks gestation)			
	Vitamin B6 (2.6mg)	73	60		WIC group women initially lighter than control women had caught up with control women by 36 weeks gestation			
	Phosphorus, Vitamins A, B1, B2, B3, B12 and C were all 95%-180% of RDA at baseline and follow-up in both WIC and control groups. RDA for pregnant women aged 19–22 years old				No clinical (or statistically) significant difference in haemoglobin at baseline or follow-up. Data not shown			
					*Adjusted for duration of gestation, conception weight and 35 other maternal characteristics as above			
	*Adjusted for duration of gestation, initial intake, 35 other maternal characteristics including smoking status, age, race, family income and size, woman and partner′s educational and employment status, social security benefits and program participation							
	181 of the 711 controls had enrolled in WIC before follow-up. The diet intake data for this group were analysed separately and were similar to the WIC intervention group							
	No difference in nutrient intake for groups at baseline							
**Rush 1988c,d**[45,46]	Food purchases*†							
	Mean (SD)	I	C	p				
	Total expenditures, $							
Standard WIC program	Recall	48.28	52.07 (33.34)	<0.001				
Pregnant women	Diary	61.20	62.85 (39.44)	NS				
	Groceries, $							
	Recall	38.30	39.95 (22.97)	<0.05				
	Diary	50.50	49.15 (35.68)	NS				
	Meals away from home, $							
	Recall	3.84	4.94 (6.44)	<0.001				
	Diary	10.93	13.69 (16.20)	<0.001				
	* Adjusted for family size, income, ethnicity, presence of father in household, maternal education, amount of food stamps and free school meals, number of guests and baseline expenditure							
	†Baseline food expenditures differed with WIC families spending significantly less on total food, groceries and meals away from home							
**Pehrsson 2001**[43]	**Dietary intake**				**Maternal outcomes**			
	Iron No data presented. No significant differences between groups. All intakes <74% RDA (15mg/day)				Mean (SD)	I	C	p
					Haemoglobin, mmol/L*	8.01 (0.82)	7.63 (0.82)	<0.05
Standard WIC program continued for 6 months post-partum					g/dL	12.8 (1.31)	12.2 (1.31)	
	Vitamin C No data presented. No significant differences between groups. All intakes >150% RDA (60mg/day)				Anaemia, % *	17	51	<0.05
					(Hb<7.45mmol/L or 12g/dL)			
Postpartum women	Biomarkers*				*Results at 6 month follow-up			
	Mean (SD)	I	C	p				
	Ferritin, ug/L	36 (20.1)	35 (20.3)	NS				
	Transferrin receptor, mg/L	6.1 (2.1)	6.5 (2.1)	NS				
	*Results at 6 month follow-up							
**Currie 2008**[49]					**Perinatal outcomes**			
					Change in low birth weight			
					% (SD)	All parity	First birth	Teen mum
Standard Food Stamp program					White	−0.014 (1.05)	0.062 (1.00) (0.92)*	0.27
Pregnant women					Black	0.47 (1.64)	0.26 (1.43) (1.58)	0.175
					*p<0.05			
					Fertility outcomes*			
					% increase in births	All parity	First birth	Teen mum
					White	3.0	13.0	6.9
					Black	12	9.0	24.6
					*Statistically significant for blacks in all categories and white first births and teen births			
**Hoynes 2009**[18]					**Perinatal outcomes**			
Standard WIC program					Mean	All births	WIC births	
Pregnant participants					Birth weight, g	3316		
					Change in birth weight, g	2.7	29 (estimated)	
					Low birth weight, %	7.2		
					Change in low birth weight	0 (−0.0784- 0.0784)		
					%, 95% CI			
					Fertility rates No statistically significant difference after WIC introduction			

**Table 3:** Nutritional and health outcomes associated with comprehensive food subsidy program for non-pregnant adults

**First Author, Year Intervention, Participants**	**Nutritional outcomes**			**Health outcomes**
**Ni Mhurchu 2010**[30]	Food purchases*			
12.5% discount on healthier foods at point-of-sale (PD)	Change in foods/nutrients at 6 months (PD- no PD)			
	Mean (95%CI)	PD	p	
	Foods, kg/week			
or tailored nutrition education (NE) or both compared to no intervention	All foods	0.90 (0.29-1.52)	0.004	
	All healthier	0.79 (0.43-1.16)	<0.001	
	All less healthy	0.07 (−0.15-0.29)	0.56	
Main household shoppers >18 years	Healthier F&V	0.48 (0.21-0.75)	<0.001	
	Healthier meat	0.06 (0.02-0.11)	<0.001	
	Healthier dairy	0.21 (0.10-0.31)	<0.001	
	No change was noted in intake of saturated fat (primary outcome), energy density or any other macronutrients			
	There were no effects of tailored nutrition education on food purchases at 6 months.			
	Change in foods/nutrients at 12 months (PD- no PD)**			
	Mean (95%CI)	PD	p	
	Foods, kg/week			
	All foods	0.37 (−0.26-1.00)	0.25	
	All healthier	0.38 (0.01-0.76)	0.045	
	All less-healthy	0.05 (−0.18-0.27)	0.67	
	Healthier F&V	0.28 (0.00-0.56)	0.05	
	Healthier meat	0.03 (−0.01-0.08)	0.15	
	Healthier dairy	0.06 (−0.04-0.18)	0.21	

*Adjusted for baseline values, ethnicity, income, age, sex.

**The 12 month follow-up was 6 months after intervention ended.

**Table 4:** Nutritional and health outcomes associated with comprehensive food subsidy programs for children

**First Author, Year Intervention, Participants**	**Nutritional outcomes**	**Health outcomes**			
**Gunnell 2000**[39]		Growth outcomes*			
Intervention included either daily school feeding (SF) or family food packages weekly (FP) and two control groups with no food subsidy- families and school students		Mean, (SD)	I	C	p
		Height increase, cm	6.10(1.50)	5.56(1.45)	<0.0001
		Leg length increase, cm	4.98 (2.07)	4.87(2.25)	0.0006
		Trunk increase, cm	1.12 (2.17)	0.69 (2.23)	0.17
		
		Stature increases by method of feeding,†			
Children aged 2–14 years		Mean (95%CI)	SF	FP	
		Change in height (I-C), mm	6.7 (2.7-10.6)	2.0 (−0.7-4.8)	
		Change in leg length (I-C),mm	7.2 (1.9-12.4)	2.3 (−1.5-6.2)	
		**Mortality**			
		Hazard ratio I vs C (95%CI)		p	
		All causes	1.15 (0.7,1.7)	0.46	
		CHD	1.62 (0.8,3.5)	0.21	
		Cancer	0.69 (0.4,1.4)	0.30	
		Non-smoking			
		related cancer	0.59 (0.2,2.0)	0.59	

* Adjusted for initial anthropometry and location.

†Adjusted for initial anthropometry, location, age, sex, duration of follow-up, household food expenditure, diet intake, family size, social class.

**Table 5:** Nutritional and health outcomes associated with fruit and vegetable subsidy programs

**First Author, Year Intervention, Participants**	**Nutritional outcomes**					**Health outcomes**			
**Burr 2007**[37]	**Dietary intake**								
	Net % consuming more fruit								
Free home-delivered orange juice using vouchers(V) nutritional advice promoting fruit and fruit juice (A) or standard care during prenatal care period (C)	Mean	V	A	C					
	Apples	2.2	−13.2	−2.7					
	Oranges	−2.2	−21.1	−11.8					
	Bananas	−17.4	−7.9	−29.7					
	Fruit juice	34.8*	−7.9	−24.3					
	*Voucher group net % significantly greater than advice or control (p<0.05)								
Pregnant women	**Biomarkers**								
	Mean (SD)								
	β-carotene change, ng/ml								
	V	A	C	p					
	n=39	n=37	n=42						
	35.6(77.2)		−20.2(43.3)	<0.0001					
		2.7(65.5)	−20.2(43.3)	0.435					
**Herman 2006 & 2008**[32,40]	**Dietary intake**								
Standard WIC program plus $10 voucher weekly for F&V from local supermarket (SM) or farmer′s market (FM)	Mean	SM	FM	C	p				
	F&V intake,								
	serves/4186kJ/day								
	End of intervention	4.1	3.9	3.0	F=9.75, p<0.001				
Post-partum women >18yo**-** all WIC participants	6 mths post-intervention	4.0	4.0	3.1	F=6.66, p=0.001				
	Vegetable intake, serves/4186kJ/day								
	End of intervention	2.3	2.1	1.5	F=11.0, p<0.001				
	6 mths post-intervention Data not shown F=−0.59, p=0.02								
	Fruit intake								
	Data not shown, no significant differences between SM, FM and control								
**Kennedy 2009**[41]	**Dietary intake**					**Anthropometric and cardiovascular outcomes**			
	Change in intake at six months					Change at six months			
Free fruit and vegetables $10/week with recipes from mobile store at community centre with monthly nutrition/cooking sessions and anthropometric assessment	Mean (SD)	I	C	p		Mean (SD)	I	C	p
	Energy, kcal/day	−456 (1032)	−636 (1326)	0.48		Weight, kg	−2.0 (3.2)	1.1 (2.0)	<0.001
	Fiber, g/day	1.7 (5.7)	−4.3 (19.7)	0.03					
African-American women	Fruit, serves/day	1.0 (1.7)	0 (1.2)	0.02					
	Veg, serves/day	0.9 (1.2)	−0.2 (1.8)	0.002		BMI, kg/m [2]	−0.7 (1.2)	0.4 (0.8)	0.001
						Waist size, cm	−0.5 (5.3)	1.9 (3.7)	0.12
						Syst BP, mmHg	−2.3 (13.0)	−1.4 (12.3)	0.14
						Dias BP, mmHg	0.8 (8.1)	0.1 (9.3)	0.68
						**Self-rated health**			
						General health and quality of life were rated across multiple domains. Trends generally favoured intervention group, but only self-esteem (p=0.03) and emotional role (p=0.04) improved significantly			

The outcomes assessed were dietary intake including fruit and vegetable intake using validated measures, biomarkers, anthropometric and growth parameters and perinatal outcomes including mean birth weight and % low birth weight. The results are presented in categories based on these outcomes: fruit and vegetable intake, other dietary intake and biomarkers, perinatal outcomes and other health outcomes.

### Diet and nutrition related outcomes

Nine of the studies (6 WIC [32,40,42,43,46-48] and 3 other studies [30,37,41]) reported participants′ nutritional status or dietary intake. Six of these studies reported dietary intake information using either 24 hour recalls [32,47,48] or food frequency questionnaires [37,41,43]. Two of the studies reported food purchases [30,46], with the more recent study using electronic shopping data [30]. Four studies reported on biomarkers of nutritional status [37,42,43,48], with three studies reporting on both biomarkers and dietary intake [37,43,48].

### Health outcomes

Health outcomes were reported by ten of the studies (seven WIC [18,38,42-44,47,48] and three other studies [39,41,49]), with all the WIC studies reporting pregnancy-related outcomes. Four of these studies reported health outcomes together with dietary intake measures [41,43,47,48]. Only the three ITS studies reported pregnancy-related health outcomes without any nutritional status measures [18,44,49].

### Impact of food subsidy programs

#### Fruit and vegetable (F&V) intake

Three RCTs [30,37,41] and one CBA study [32] reported on F&V intake/purchases. There were consistent and significant increases in the measures of F&V intake in these studies (Tables 3 and 5). Only the supermarket price discount RCT was a comprehensive food subsidy. In this study, Ni Mhurchu et al. showed an increase of 0.48kg/week (95% CI 0.21-0.75) of healthier F&V purchased, which was an 11% increase from baseline after 6 months of a 12.5% supermarket price discount intervention. At 12 month follow-up (6 months after intervention) increased purchases of healthier F&V (0.28kg/week 95% CI 0–0.56) persisted, but was attenuated [30]. This was a large RCT (n=1104) that met most quality criteria and with a low risk of bias, however, there was no change in the primary outcome, saturated fat purchases, in this study (Table 3).

The remaining three studies assessed limited subsidies of either F&V or fruit juice. None were assessed to have a low risk of bias. In the community centre F&V RCT (n=40), Kennedy et al. reported that overweight women who received US$10/week of F&V for six months increased mean consumption of fruit by 1.0 serves/day (95% CI 0.1-1.9, p=0.02) and vegetables by 0.9 serves/day (95% CI 0.3-1.5, p=0.002) compared to controls, whose intake was unchanged [41]. Burr et al. assessed the impact of free home-delivered fruit juice and/or nutrition education throughout prenatal care on the overall fruit intake of pregnant females in a RCT (n=190) in the UK [37]. Among those who received subsidised juice, there was a net increase of 34.8% in the proportion consuming more fruit juice, while the intake of fruit juice declined among the control (−7.9%) and nutrition education groups (−24.3%). Herman et al. assessed the impact of an additional US$10/week fruit and vegetable subsidy for six months for WIC participants in California at either a supermarket or farmer′s market in a CBA study [32,40]. Participants receiving subsidised F&V consumed 4.1 serves/4186kJ/day (Supermarket) or 3.9 serves/4186kJ/day (Farmer′s market) while controls consumed 3.0 serves/4186kJ/day (F=9.75, p<0.001). This increase persisted in both groups 6 months after the intervention (Table 5).

#### Other dietary outcomes and biomarkers

Four comprehensive food subsidy programs reported on other dietary outcomes: the supermarket price discount RCT [30] and three WIC CBA studies [43,47,48] (Table 2). In the supermarket price discount RCT, the only study with a low risk of bias, Ni Mhurchu et al. found that a 12.5% price discount significantly increased purchases of total (0.90 kg/week 95% CI 0.29-1.52) and healthier food (0.79kg/week 95% CI 0.43-1.16) after 6 months [30]. The most comprehensive estimates of dietary intake were from the National WIC evaluation which assessed 6563 pregnant women [47]. This study reported that the intake of most macronutrients and micronutrients were 10-20% (p<0.05) higher among WIC participants compared to controls, including four of the five nutrients targeted by the WIC program: protein, calcium, iron and vitamin C. The smaller CBA studies showed variable results: Bailey et al. found intake of folate was higher, iron and B6 the same, and protein lower among prenatal WIC participants; [48] Pehrsson et al. found no difference in iron intake between postnatal WIC participants and controls [43].

Four studies reported the impact on nutrition-related biomarkers; three were comprehensive food subsidies (all WIC studies) (Table 2) and one fruit and vegetable subsidy (the fruit juice RCT) (Table 5). None of these studies were assessed to have a low risk of bias. In the WIC program RCT, Metcoff et al. found non-significant increases in β-Carotene (10% p=NS) and iron-binding capacity (27%, p=NS) among pregnant WIC participants after adjustment for duration of gestation, baseline level and interval between measurements [42]. The two smaller WIC CBA studies showed variable effects on biomarkers. Pehrsson et al., who found no difference in iron intake, also found that ferritin and transferrin receptors were not significantly different in postnatal WIC participants and non-participants [43]. Bailey et al., who reported higher folate intake and no difference in iron and vitamin B6 intake, showed that serum vitamin B6 and transferrin saturation were significantly higher in WIC participants, however, serum iron showed no difference and both red cell folate and serum folate were lower in WIC participants (p<0.05 only for serum folate) [48]. In the fruit juice RCT, Burr et al. found fruit juice vouchers increased β-carotene in pregnant recipients (mean (SD) 35.6 (77.2) ng/ml) compared to a decrease in controls (−20.2 (43.3) ng/ml p<0.0001) [37] (Table 5). This was consistent with the increase in the proportion consuming fruit juice observed in the same study.

#### Perinatal outcomes

**Figure 2:**
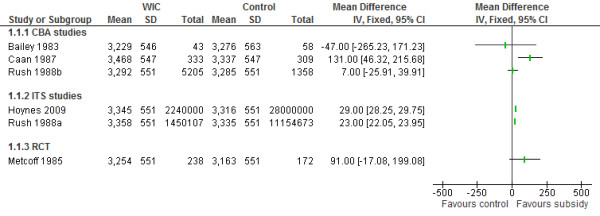
Forest plot of the impact of food subsidy programmes on birth weight in grams (WIC studies).**SD is missing for Rush 1998b, Hoynes 2009, Rush 1988a and Metcoff 1985 and average SD from available studies is used as an estimate.

Six WIC studies reported on mean birth weight and low birth weight <2500g (LBW) proportion- 1 RCT [42], 2 ITS [18,44] and 3 CBA studies [38,47,48] (Table 2). The Food Stamp Program ITS study [49] also reported on LBW (Table 2). All of these were comprehensive food subsidy programs. The three ITS studies were the only ones among these studies assessed to have a low risk of bias. As sufficient data were available, Cochrane Review Manager 5 [51] was used to produce a forest plot, with weighted mean differences and 95% confidence intervals for mean birth weight in the WIC studies. The heterogeneity of study designs and missing data precluded the reporting of a summary statistic for mean birth weight. However, overall there would appear to be a small increase in mean birth weights as reflected in the forest plot of this outcome for WIC studies (Figure 2) and a non-significant trend to decreased rate of low birth weight among WIC participants.

The two WIC ITS studies reported on birth weight trends during the 1970s when the WIC program was progressively implemented in the USA. Hoynes et al. found a significant 2.7g increase in mean birth weights in the total sample attributable to WIC (Mean birth weight 3316g), which they estimated as a 29g increase in infants of WIC participants, based on WIC participation rates [18]. Rush et al. estimated that WIC participation increased mean birth weight by 23g (mean birth weight 3335g p<0.05) [44]. The WIC RCT found a non-significant 91gram increase in mean birth weight of infants whose mothers received WIC during pregnancy (WIC 3254g vs. Control 3163g p=NS) after adjusting for maternal weight [42]. The National WIC evaluation CBA study found a non-significant 7 gram increase in mean birth weight (WIC 3292g vs control 3285g p=NS) [45,47]. In the two smaller CBA studies, Bailey et al. showed mean birth weight was 47g lower in WIC infants (although statistically non-significant) (WIC 3229g vs control 3276g p=NS) [48] and Caan et al. found mean birth weight of WIC infants 131g higher (WIC 3468g vs control 3337g p=0.003) [38]. Two studies reported on the impact of prenatal WIC participation among smokers. In the WIC RCT, the mean birth weight of infants of WIC participants who smoked (>10 cigarettes/day) was significantly higher than those of controls who smoked (WIC 3235g vs control 3059g, p=0.017) [42]. Bailey et al. found that whereas there was significantly lower mean birth weight among smokers compared to non-smokers in the control group, the WIC smokers had (non-significantly) higher mean birth weight than WIC non-smokers [48].

The three ITS studies showed no significant impact on LBW proportions. Hoynes et al. reported no change in the proportion of low birth weight due to WIC (% LBW=7.2%) [18]. Rush et al. reported a non-significant decrease in LBW proportion of 0.43% among WIC participants (Total sample % LBW=6.84%) [44]. In the Food Stamp study, Currie et al. found no significant changes overall in low birth weight in California after the introduction of the Food Stamp Program in the 1960s [49]. In the National WIC evaluation CBA study, a non-significant decrease of 1.13% in the proportion of LBW was reported (WIC 5.62% vs control 6.75% p= NS) [47]. There were non-significant changes in % LBW in the two smaller WIC CBA studies and the RCT, Bailey et al. (WIC 5% vs control 10%, p=NS) [48], Caan et al. (WIC 3.2% vs control 5.1%, p=0.08) [38] and Metcoff et al. (WIC 8.7% vs control 6.9%, p=NS) [42]. Only the two National WIC evaluation studies by Rush et al. [44,47] and the Caan et al. study [38] adjusted LBW proportion for gestational age.

#### Other health outcomes

Four comprehensive food subsidy programs- all WIC studies- reported maternal anthropometry and/or haematological parameters. [38,42,43,47] Only the CBA study by Pehrsson et al. [43] was assessed to have a low risk of bias. Three CBA WIC studies reported maternal haemoglobin (Hb) levels (Table 2): Rush et al. in the national WIC evaluation found a non-significant 0.06g/dL increase in Hb among prenatal WIC participants [47]. Pehrsson et al. and Caan et al. found postnatal WIC participation significantly increased Hb by 0.61g/dL (5%) [43] and 0.29 g/dL (2.4%) [38] compared to controls respectively. Three studies also reported on maternal anthropometry (Table 2). In the WIC RCT, Metcoff et al. found prenatal WIC participants had greater weight gain during pregnancy (p=0.19) [42]. Rush et al. found that WIC participants started with significantly lower weights in early pregnancy, but had identical weight in late pregnancy to controls [47]. Caan et al. found maternal de Quetelet′s index decreased significantly during pregnancy [38].

Only the 1930s family food packages study in the UK reported on health outcomes in children. In this study, Gunnell et al. found that food subsidies increased children′s height and leg length significantly, however, there was no significant impact on mortality after 60 years follow-up (Table 4) [39].

None of the studies of pregnant or non-pregnant adults reported on episodes of illness, mortality or morbidity or health service/hospital attendances.

#### Adverse effects

The only possible adverse effects reported were significantly higher fertility rates among teenage mothers in the Food Stamp Program study [49] and significantly lower total food and groceries expenditure (based on recall but not diary method) among WIC participants compared to controls in the National WIC evaluation CBA study [46] (Table 2).

## Discussion

This review found a limited number of rigorous studies have been undertaken to investigate the impact of food subsidy programs on participants′ nutritional intake and health outcomes. However, there have been an increasing number of high quality studies reported in the last five years. The targeted F&V subsidies with nutrition education were able to increase F&V intake by 1–2 serves/day in women. The National WIC evaluation CBA study showed increases of 10-20% in measured nutrients in pregnant women. Studies of other comprehensive and fruit and vegetable subsidies showed similar improvements in nutrient intake, biomarkers or food purchases. Thus, there are measurable improvements in nutrition, even though they cannot be precisely quantified in this review. Evidence of the link between improvements in nutritional status and in health outcomes is predominantly from perinatal outcomes of the WIC program. There is consistent evidence of a small but significant increase in mean birth weight from the two WIC ITS studies, although there is no evidence of significant changes in the proportion of low birth weight. There is suggestive evidence that the WIC program may have a more substantial impact on the mean birth weight of infants whose mothers are regular smokers.

This review was conducted according to the protocols recommended by the Cochrane Collaboration [26]. A comprehensive search strategy and screening process makes it is likely that relevant published studies were identified. Moreover, this review focussed on studies with robust research designs, increasing the likelihood that any positive effects could be attributed to the interventions [52].

The inclusion of 10 non-randomised studies increases the possibility of selection bias and residual confounding as alternative explanations for the positive impacts of food subsidy programs discussed above. In addition, only one of the four RCTs was assessed as having a low risk of bias. Thus, the overall quality of the evidence is limited and the findings should be interpreted cautiously. The heterogeneity of both study design and outcomes also prevented meta-analysis and limited the precision of estimates of effect. The studies assessed to have a low risk of bias (one RCT [30], 3 ITS studies [18,44,49] and one CBA study [43]) found a range of improvements in food purchases, nutritional biomarkers and perinatal outcomes that have significance for the health of the population. As with other complex public health interventions, there may be practical and ethical limitations on undertaking RCTs, and it will remain important to assess all available high quality evidence with due attention to the limitations of the overall evidence base.

There were also limitations in individual studies. Most studies reported multiple nutritional outcomes without specifying a primary outcome, which increases the likelihood of significant associations occurring by chance [53]. Much of the dietary intake data was based on self-report, which has well recognised limitations due to imprecision and potential recall biases [54]. Biomarkers, such as carotenoids and vitamin C, and electronic shopping data are more precise and objective measures of dietary behaviour with different potential biases [55,56]. Thus, greater use of these measures has the potential to complement self-reported intake data. There were limited data reported on the health and nutritional outcomes for children and no data for men. WIC studies have reported nutritional improvements in children [57,58], but more rigorous study designs would strengthen these conclusions. An exclusive focus on women and young children in food subsidy programs does not reflect completely the social dynamics that shape dietary behaviour [59]. Research involving whole families would increase the understanding of the wider impacts of food subsidy programs. Although only limited adverse effects were reported in these studies, this is more likely to indicate the absence of evidence rather than evidence of no adverse effects. There should be improved efforts to identify all impacts of food subsidy programs.

The findings of a positive impact on birth weight in this review contrast with those of D′Souza et al. [24],. who concluded that the WIC program did not have a significant impact on birth weight or the likelihood of low birth weight, except possibly for smokers. The conclusions in this review of WIC′s impact on birth weight were based particularly on the two ITS studies [18,44] (assessed as low risk of bias) that were not included in the D′Souza et al. [24] review. However, the modest impact on perinatal outcomes supports their suggestion that food subsidy programs should focus on improving nutritional status with the aim of improving longer term health outcomes.

In this review, only Gunnell et al. [39] reported long-term health outcomes, finding no mortality benefit from a 12 month childhood food subsidy program. This 12 month program was the longest intervention included in the review. However, a 10-20% increase in key nutrients or 1–2 serves of F&V/day have the potential, if sustained, to decrease rates of non-communicable diseases, including ischaemic heart disease, cancer and diabetes, and ultimately mortality. Prospective longitudinal studies have confirmed the association between healthier nutrition and better health outcomes [60-62]. For example, it is estimated that for each additional serve of F&V consumed, there is a 4% decrease in the risk of coronary heart disease [62,63]. These reductions in chronic disease prevalence were found in a relatively affluent and well-nourished cohort and the benefits may be greater in a more disadvantaged group.

The evidence of sustainable impacts on dietary behaviour as a result of food subsidy programs in both adults and children is limited. Only two included studies reported on follow-up post-intervention- both after subsidies of six months duration- and found persistence of significantly increased F&V intake [32] and healthier food purchases [30] at 12 months respectively (though 50 % less than while receiving the food subsidy in the latter study [30]). The WIC program provides food subsidies for longer (up to age five for children), however, Rush et al. [64] found that the dietary intake improvements in children were only seen in current WIC participants. The optimal duration and design of food subsidy programs is unlikely to have a simple answer given the complex influences on dietary behaviour [65] but remains important given the link between lower socio-economic status and less healthy nutrition [3,66].

Longitudinal controlled studies of sufficient duration which quantify the extent of any reductions in chronic diseases from specified improvements in nutrition would build the strongest possible evidence base for food subsidy programs. However, most evaluation research of food subsidies is likely to focus on measures of nutritional intake as markers of improvement. Future nutrition intervention evaluations should use robust validated nutritional intake measures reported in a standardised format (eg. repeated 24 hour recalls) to enable comparisons with other studies including meta-analysis [67]. As discussed, the use of objective measures, including biomarkers and electronic shopping data, should be considered as alternatives to self-reported intake data where feasible. Assessment of the overall diet, using principle component analysis or comparison with dietary indices, is another alternative that provides realistic data to complement other dietary intake assessments [68,69] and addresses the inherent limitation of significance testing when evaluating multiple outcomes, discussed above.

Further economic analyses based on high quality outcome studies would provide complementary data to help determine whether new or amended food subsidy program represent an effective investment within a country. Such analysis would facilitate comparison of potential cost-savings in the health system with the tangible upfront public funding required. In the USA, positive cost-benefit analyses are part of the explanation for the investment in food subsidies [70,71]. However, D′Souza et al. [24] concluded that the WIC economic analyses were of poor quality. An evaluation of the Healthy Start program in the UK [72,73] was limited by the long-standing commitment to welfare food entitlements which prevented allocation using randomisation. This will reduce the strength of economic analyses based on such data.

Non-cash programs, particularly the food subsidy programs, are central to the social security system for low income people in the USA. For countries with their own specific social security systems, the outcomes of food subsidy programs may be quite different. The impact may depend on whether they reduce existing benefits or income or represent an additional investment to improve the health status of the population or decrease inequality. Modelling studies suggest that healthy food subsidies in combination with taxation of less healthy foods could have a larger impact on the nutrition of those on low incomes [74]. However, the provision of a food subsidy could allow increased spending of other income on less healthy foods, cigarettes or alcohol. The negative health impacts could outweigh the positive nutritional impacts of the food subsidy, as was suggested in one unhealthy food taxation modelling study [31]. The decision about which foods to subsidise (or tax) is important. Although programs which subsidised only fruit, vegetables and/or juice showed increased intake of targeted foods in this review, it is important to focus on the impact on overall dietary intake in the design and implementation of a food subsidy program. The design of any food subsidy program should also include consideration of how to mutually reinforce other public health initiatives, including social marketing, school nutrition programs and healthy lifestyle programs. In the USA, WIC promotes preventive health activities effectively and links well with health services [75], although operating as an independent service funded by the US Department of Agriculture.

## Conclusions

This review identified limited high quality evidence of the impacts of food subsidy programs on the health and nutrition of adults and children in high income countries. This evidence, predominantly from studies of the WIC program in the USA, suggests that female participants have 10-20% increases in targeted nutrients and foods due to these food subsidy programs. Associated improvements in perinatal outcomes were limited and most evident in women who smoked during pregnancy. Thus, food subsidy programs for pregnant women and children should aim to improve nutritional status in the longer term. The improved intake of targeted foods such as fruit and vegetables could potentially reduce the rate of non-communicable diseases in adults, if the changes in diet are sustained. There have been virtually no adverse effects of food subsidy programs reported. Further prospective data is needed to confirm that food subsidies produce sustainable improvements in dietary intake and document any adverse effects. Evaluation of new initiatives should include controlled trials where possible and economic analyses to provide evidence of effectiveness in a local context.

## Competing interests

The authors have no competing interests to declare.

## Authors’ contributions

All authors conceived the review. APB, KO, JB, HE and PM participated in the design of the review. APB and JB selected the studies. APB extracted the data and drafted the manuscript with input from all authors. All authors read and approved the final manuscript.

## Funding

The conduct of this review was supported by the National Health and Medical Research Council (PhD scholarship ID No.520681 and Program Grant 320860).

## Pre-publication history

The pre-publication history for this paper can be accessed here:

http://www.biomedcentral.com/1471-2458/12/1099/prepub

## Supplementary Material

Additional file 1: Table 3 Summary of risk of bias assessment on each relevant item for included studies. **Table 6 **Summary of risk of bias assessment on each relevant item for included studies1. Click here for file
